# P-239. A Mixed-Methods, Formative Evaluation of Advance Care Planning Documentation in People Living with HIV Aged 50 and Older

**DOI:** 10.1093/ofid/ofaf695.461

**Published:** 2026-01-11

**Authors:** Mary R Bartkus, Elizabeth Jadovich, Sergio Romero, Samantha Roche, Athina Schmidt, Laura Mitten, Irina Vovnoboy, Archana Asundi

**Affiliations:** Boston Medical Center, Boston, MA; Boston Medical Center, Boston, MA; Boston Medical Center, Boston, MA; Boston Medical Center, Boston, MA; Boston Medical Center, Boston, MA; Boston Medical Center, Boston, MA; Boston Medical Center, Boston, MA; Boston Medical Center, Boston, MA

## Abstract

**Background:**

People living with HIV (PWH) are living longer due to effective antiretroviral therapy. As they age, PWH encounter increased difficulties with aging compared to the general population. With increased rates of chronic co-morbidities and accelerating aging, it is important that PWH be given the opportunity to engage in advance care planning (ACP). However, there remains a significant gap in ACP preparation among this population.

**Figure:**
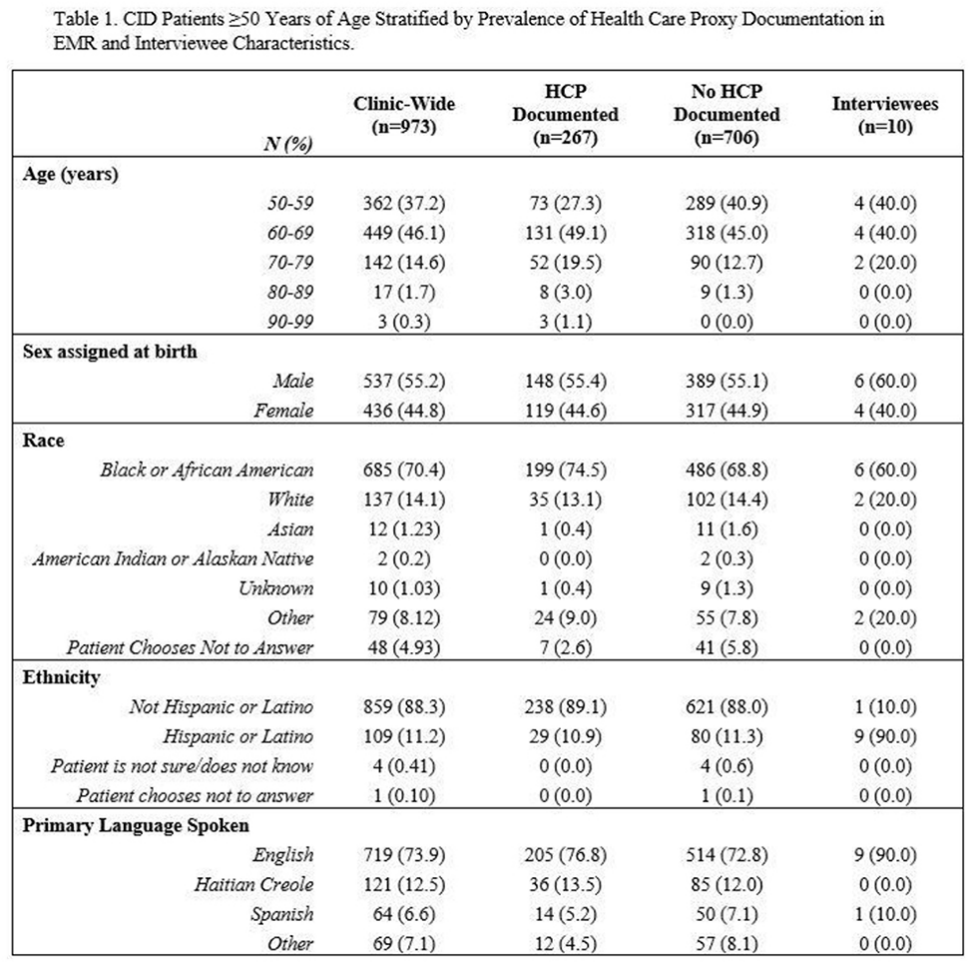

**Figure:**
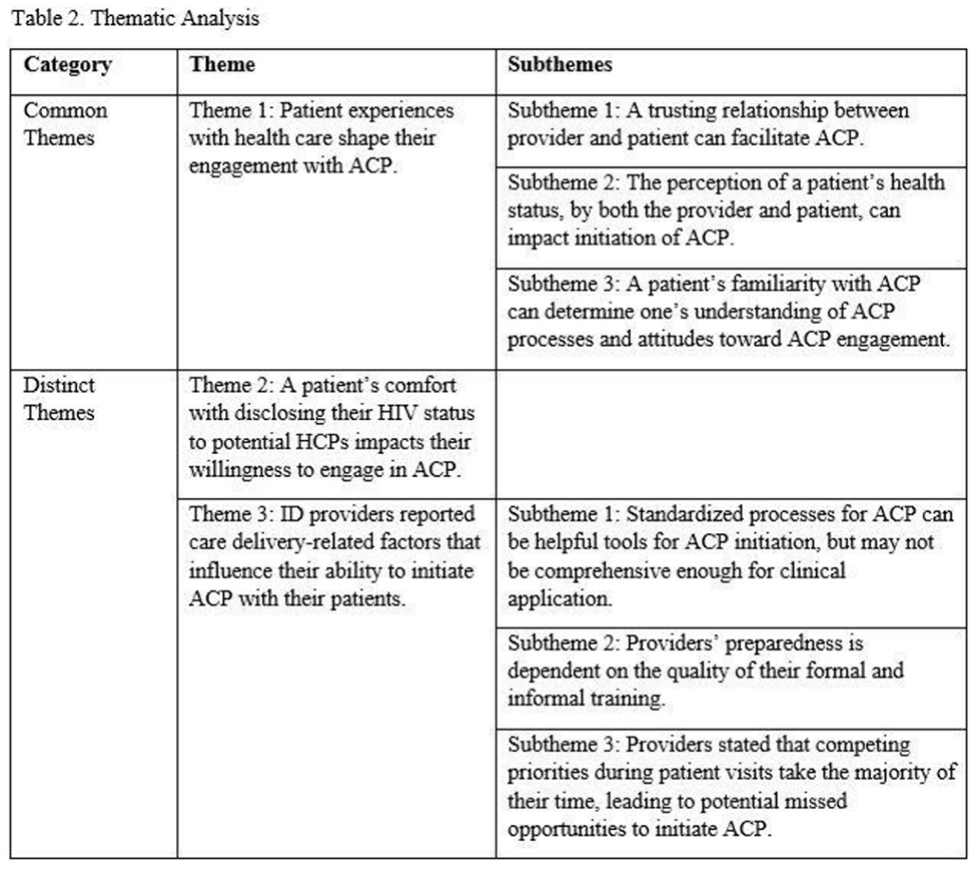

**Methods:**

A retrospective chart review of PWH ≥50 years (N=973) was conducted to generate descriptive statistics and evaluate prevalence of ACP documentation in the clinic. Then, CID providers (N=5) and patients (N=10) were interviewed regarding their ACP experiences. Interviews were coded using an inductive, open coding process. For analysis, emerging themes and subthemes were identified to describe the perceptions of ACP among interviewees.

**Figure:**
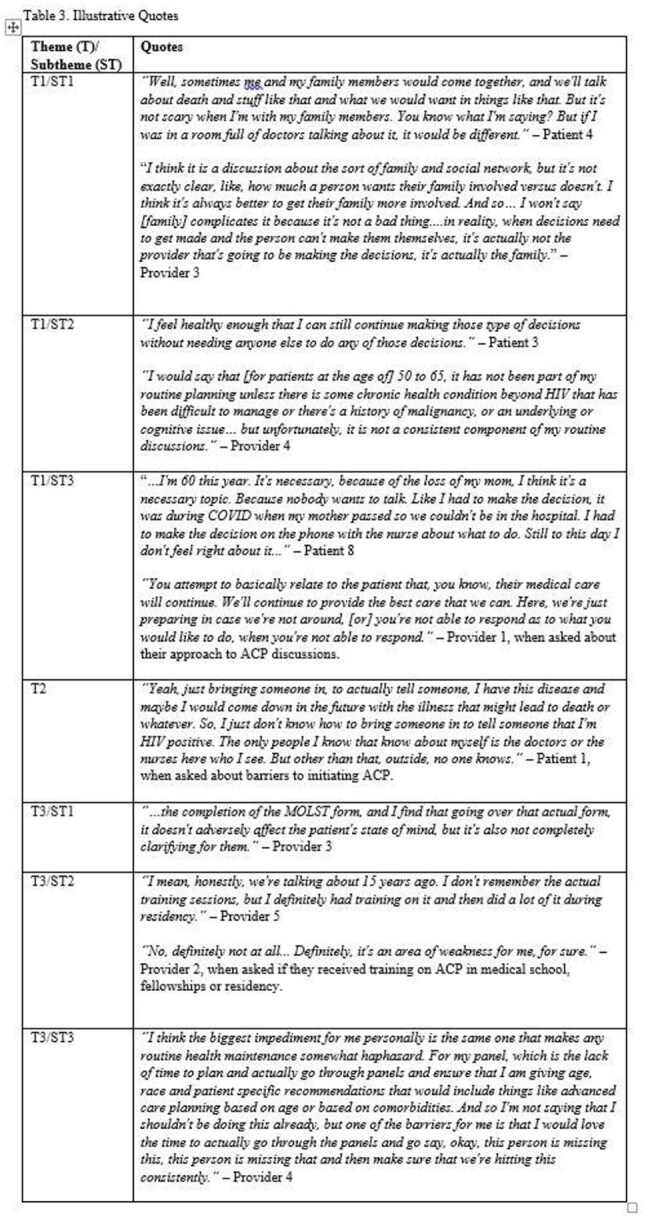

**Results:**

73% (N=706) of patients in CID had no Health Care Proxy (HCP) documented in the EMR with no clear sociodemographic differences (Table 1).

Interviews revealed individual social comfort with ACP discussions, health status, and prior knowledge were the most significant factors that contributed to one’s willingness to initiate ACP.

Patient interviews confirmed the concept of disclosing one’s HIV status to their support network as a factor when appointing a HCP.

Providers discussed the workflow and training associated factors that impact ACP initiation with their patients including time, quality of resources, and depth of training (Tables 2, 3).

**Conclusion:**

There are many factors associated with ACP initiation, many of which are tailored to one’s specific experiences. The study team intends to use these findings to inform a clinic-wide intervention that would promote collaborative approaches to ACP between patients and providers, such as educational initiatives and tailored resources.

**Disclosures:**

Archana Asundi, MD, DayZero Diagnostics: Grant/Research Support|Gilead Sciences: Advisor/Consultant|Gilead Sciences: Grant/Research Support|Theratechnologies: Grant/Research Support|Viiv Healthcare: Grant/Research Support

